# Guy's Hospital Reports. No. I. January, 1836

**Published:** 1836-04

**Authors:** 


Art. XIX.—*Guy's Hospital Reports.* No. I. *January*, 1836. Edited by George H. Barlow, m.a. Trin. Coll. Cam., Inceptor Candidate of the Royal College of Physicians; and James P. Babington, m.a. Trin. Coll. Cam., Member of the Royal College of Surgeons.—8vo. pp. 188. *London*, 1836.

*St. Thomas's Hospital Reports.* By John F. South, Assistant Surgeon. No. II. *January*, 1836.—8vo. pp. 123.

The medical public, it will be perceived, is likely to be abundantly supplied with authentic clinical reports. Both of the above-named publications contain several instructive cases. The first number of the Reports of St. Thomas's Hospital was noticed in our last, and the second is at least of equal merit. The first number of the Guy's Hospital Reports is introduced by an academical exercise upon medicine and medical experience, from which however we extract the following interesting account of the institution from which the Reports proceed.

"Guy's Hospital contains within its wards a constant succession of above five hundred patients: thus amounting in the year to between three and four thousand; not admitted at the recommendation of governors, but selected by the physicians and surgeons, as those whose diseases are the worst and most acute, or the most interesting and instructive, amongst a concourse of patients who present themselves upon the regular days of admission.

"There are also in this hospital two spacious wards; the one containing twenty-three males, and the other eighteen females, set apart for the purpose of clinical instruction. Into these wards are received such patients as the clinical physician may deem most fit for the purpose of illustrating disease. There is also an eye-infirmary, consisting of two wards, containing each about fourteen beds: and a portion of one of the wards is appropriated to females labouring under uterine disorders, and who are selected by the obstetric physician as those whose cases seem most available for the purpose of clinical instruction in this highly interesting class of diseases. The annual number of accidents admitted amounts to nearly six hundred; and thus are afforded opportunities of witnessing the various forms of injury, to which the different working classes in this metropolis must be continually exposed; and these, as well as cases occurring amongst the other surgical patients, necessarily give rise to very many and various operations. Very curious and highly-instructive forms of disease are, moreover, to be found amongst the out-patients: who, to the number of no less than fifty thousand, in the course of the year present themselves, upon stated days, to be prescribed for by the physicians and surgeons: and there is, connected with the hospital, a lying-in charity, by means of which, about five hundred women are annually delivered at their own habitations.

"From these various departments, then, of this vast and complete institution, every form of disease, which can occur in this climate, is from time to time presented to our attention, and may be watched through its successive stages, until it is either brought to a successful issue or terminates in death. The lesson, however, that may be learned from every fatal case of disease does not end with the life of the sufferers, the most important facts being often brought to light by examination after death; accordingly, facilities for pathological investigation are not wanting in this extensive institution: not only are inspections publicly performed, but reports are made, both of the appearances observed, and of the most prominent symptoms which occurred, during life. These reports are placed in the museum, not unfrequently accompanied by preparations of the parts to which they refer.

"Of this museum it is needless to speak at present, as some of our future pages will be expressly devoted to it: suffice it, therefore, to say, that although in years it may be considered in its infancy, yet, from the number and variety of its preparations, it might be supposed to be in its maturity." (*Introduction*, p. ix.)

Among the contributors of cases to this number are Dr. Bright, Dr. Addison, Sir Astley Cooper, Mr. Key, Mr. Morgan, and Mr. Bransby Cooper; and it is almost superfluous to say that the cases are, generally speaking, well selected and well related. But we cannot forbear to express our opinion that *one* hospital journal would be better than the number which may now be expected. *One* such journal, consisting of cases selected from *all* the hospitals, would be useful to innumerable readers. *Many* such journals must necessarily be made up of materials of variable value, and the business of selection will devolve on the other periodicals; by which means the circulation of the Hospital Reports will not receive adequate encouragement to ensure their continuance. Common cases, illustrated by plain practical remarks, are chiefly useful to the student in the hospital wards. The readers of Hospital Reports look for the most important cases, or classes of cases, and expect appropriate commentaries, and these would be best supplied by a general Journal of Clinical Reports, or Hospital Journal, to which both town and country hospitals might be invited to contribute. We fear we shall be considered very much prejudiced if we again allude to the frequent imperfection of the Clinical Reports in the London schools. It is curious that the first case in the Guy's Hospital Reports should present a striking example of this, even from Dr. Bright; whose high and deserved reputation will not suffer from our pointing out this instance of negligent reporting. The case is one of simple fever, protracted by irritation of the bowels, and attended by relapse. The subject, a youth of fourteen, had been ill fourteen days; and on admission is mentioned as being "greatly oppressed with symptoms of acute fever: skin very hot, and covered with miliary eruption: pulse 130, feeble, but sharp. He said he had no headach, but had a tendency to wander. His bowels were relaxed, and there was some tension of the abdomen: tongue dry, brownish in the centre, white at the edges: no cough." This is meager enough, and, we suspect, not fully correct; for two days afterward, the daily report says "abdomen less tender," although no tenderness had before been mentioned. Nor are the subsequent daily reports sufficiently full in such a case as fever, in which even minute symptoms are important. Dr. Bright's observations are practical and useful. So also the case of poisoning in the first page of the St. Thomas's Reports is very deficient in particulars. Still, with such large hospitals at command, and the assistance of so many physicians and surgeons of talents and large experience, it is evident that a quarterly hospital report from St. Thomas's or from Guy's must always be rich in clinical facts, to which practitioners in every part of the country will refer with interest.

